# Congenital subclavian-superior vena cava arteriovenous fistula with initial stenosis in an adult: a case report

**DOI:** 10.1186/s12872-020-01660-5

**Published:** 2020-08-17

**Authors:** Geng Li, Xingjian Hu, Yin Wang, Si Chen

**Affiliations:** Department of Cardiovascular Surgery, Union Hospital, Tongji Medical College, Huazhong University of Science and Technology, Jiefang Avenue 1277#, Wuhan, 430022 P.R. China

**Keywords:** Congenital arteriovenous fistula, Fistula stenosis, Adult, Case report

## Abstract

**Background:**

A subclavian-superior vena cava arteriovenous fistula is usually acquired and secondary to trauma or operations, while congenital causes are very rare. A congenital arteriovenous fistula leads to congestive heart failure soon after birth and is typically diagnosed in early infancy.

**Case presentation:**

We present an unusual case of a 21-year-old female suffering from new-onset heart failure at 20 years old who was diagnosed with a congenital arteriovenous fistula from the right subclavian artery to the superior vena cava (RSA-to-SVC) with stenosis at the proximal initial site of the fistula. The patient successfully underwent transcatheter occlusion for the fistula and had a significant improvement in symptoms at the 3-month follow-up.

**Conclusions:**

An RSA-to-SVC fistula is a very rare congenital disorder that can lead to shunt-related heart failure. If there is an indication for closure, as with the patient presented, percutaneous device closure can be considered a reasonable option.

## Background

Arteriovenous fistulas involving the subclavian artery and vein are uncommon in adults. They are usually acquired secondary to trauma or operations, while congenital causes are very rare [1]. Here, we present an unusual case of an adult patient suffering from new-onset congestive heart failure at 20 years old who was finally diagnosed with a congenital arteriovenous fistula from the right subclavian artery (RSA) to the superior vena cava (SVC) along with stenosis at the proximal initial site of the fistula, and the “proximal initial site” was the fistula entrance near the RSA.

## Case presentation

A 21-year-old girl complaining of a six-month history of progressive dyspnoea and chest pain was transferred to our centre because of heart failure, without a history of any cardiovascular diseases, injuries or operations.

On examination, there was a grade 3/6 continuous machinery murmur that was maximal between the right 2nd and 3rd intercostal region and radiated to the right infraclavicular fossa. The patient had a normal saturation value at rest in ambient air (SPO2 95%) with non-cyanotic skin colour. Chest-X-ray revealed cardiomegaly. The electrocardiogram showed sinus rhythm with a heart rate of 95 bpm and a complete right bundle branch block and right ventricular hypertrophy. The respiratory tests were not abnormal.

**Fig. 1:**
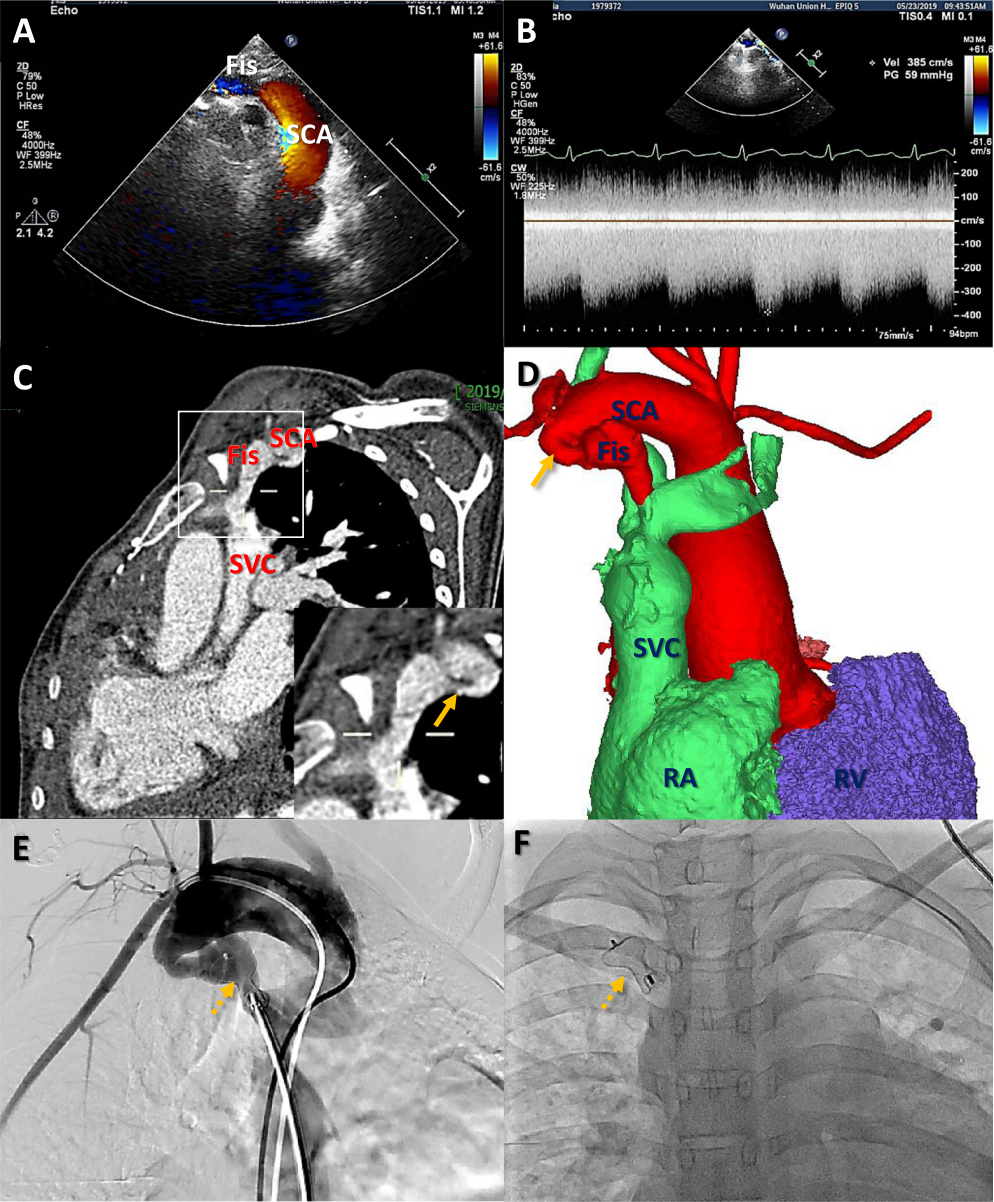
**a** Transthoracic echocardiography shows the arteriovenous fistula (Fis) between the right subclavian artery (SCA) and the superior vena cava (SVC); **b** Continuous-wave Color Doppler interrogation of the fistula shows high velocity flow signals moving from the SCA into the SVC throughout the cardiac cycle (stenosis site: velocity 385 cm/s, pressure gradient 59 mmHg); **c**, **d** Computed tomographic angiography and three-dimensional reconstruction show the fistula and obvious stenosis site (arrow); **e**,**f** The fistula is successfully closed using a 10/12 mm Amplatzer Ductal Occluder (asterisk) by the guidance of angiography (dotted arrow); RA — right atrium; RV — right ventricle

Transthoracic echocardiography showed a dilated right subclavian artery with an 8-mm fistula to the SVC and obvious stenosis at the proximal initial site of the fistula, in addition to a markedly dilated right ventricle and right atrium and mild tricuspid regurgitation (Fig. 1a). Continuous wave Doppler showed a flow signal at 2.3 m/s that was continuously moving from the RSA to the SVC with a gradient of 22 mmHg, while the highest flow rate was 3.9 m/s at the stenosis site of the fistula with a gradient of 59 mmHg (Fig. 1b). Computed tomography angiography further delineated the anatomy of the arteriovenous fistula from the RSA to the SVC and stenosis of the fistula (Fig. 1c, d).

The patient underwent transcatheter occlusion for the fistula under local anaesthesia. Briefly, a 10/12 mm Amplatzer ductal occluder was delivered and deployed from the SVC side using a 5-F H1 catheter by angiogram guidance, and an 8F sheath was used to send the occluder to occlude the abnormal fistulous connection (Fig. 1e, f and supplemental video). The post-procedure angiogram revealed a completely occluded lumen of the fistula, and the echocardiogram showed no residual shunt. The patient had an uneventful course and a significant improvement in symptoms at the 3-month follow-up.


**Additional file 1: Video.**

## Discussion and conclusions

Acquired arteriovenous fistulas in the thoracic cavity are more common than congenital arteriovenous fistulas. In the case described above, the patient had no history of trauma or previous surgeries, so it could be deduced that the arteriovenous fistula was congenital in origin. Congenital arteriovenous fistulas usually form an abnormal communication between the aorta and right heart, such as in a Valsalva fistula or coronary arterial fistula [2]. However, arteriovenous fistulas from the RSA to the SVC are rare. Awasthy et al. described an unusual case of a 4-month-old infant with a congenital arteriovenous malformation between the RSA and the SVC that was associated with SVC stenosis [3]. Balakrishnan et al. reported a case of a neonate with a congenital arteriovenous fistula from the subclavian artery to the SVC that resulted in neonatal heart failure [4]. Congenital arteriovenous fistulas from the aortic branch to the SVC often lead to congestive heart failure in newborns and infants; therefore, it is always diagnosed and treated as early as possible [5].

Surprisingly, the patient in the present case was asymptomatic for nearly 20 years after her birth, while new-onset symptoms of right heart failure had only presented in the last 6 months. She was diagnosed with a large congenital arteriovenous fistula (8 mm) from the RSA directly to the SVC for the first time. In this case, the occurrence of congestive heart failure was delayed, and it was likely due to stenosis at the proximal initial site of the fistula, which reduced the anomalous “left- to-right” shunt from the right subclavian artery to the superior vena cava.

To our knowledge, this may not be the first human case, but it is obviously the first one in the MEDLINE database to date. RSA-to-SVC fistulas are very rare congenital disorders that can actually lead to shunt-related heart failure. If there is an indication for closure, as with the patient presented, percutaneous device closure can be considered a reasonable therapy option.

## Data Availability

All data generated or analyzed during this study are included in this published article and its supplementary information files.
